# Research on Online Traceability Methods for the Causes of Longitudinal Surface Crack in Continuous Casting Slab

**DOI:** 10.3390/ma18153695

**Published:** 2025-08-06

**Authors:** Junqiang Cong, Qiancheng Lv, Zihao Fan, Haitao Ling, Fei He

**Affiliations:** School of Metallurgical Engineering, Anhui University of Technology, Ma’anshan 243032, China; mingh_cong@163.com (J.C.); 13635579157@163.com (Q.L.); linghaitao@ahut.edu.cn (H.L.); hf2573546@sina.com (F.H.)

**Keywords:** continuous casting slab, surface longitudinal cracks, causes, traceability, LightGBM-SHAP

## Abstract

In the casting and rolling production process, surface longitudinal cracks are a typical casting defect. Tracing the causes of longitudinal cracks online and controlling the key parameters leading to their formation in a timely manner can enhance the stability of casting and rolling production. To this end, the influencing factors of longitudinal cracks were analyzed, a data integration storage platform was constructed, and a tracing model was established using empirical rule analysis, statistical analysis, and intelligent analysis methods. During the initial production phase of a casting machine, longitudinal cracks occurred frequently. The tracing results using the LightGBM-SHAP method showed that the relative influence of the narrow left wide inner heat flow ratio of the mold was significant, followed by the heat flow difference on the wide symmetrical face of the mold and the superheat of the molten steel, with weights of 0.135, 0.066, and 0.048, respectively. Based on the tracing results, we implemented online emergency measures. By controlling the cooling intensity of the mold, we effectively reduced the recurrence rate of longitudinal cracks. Root cause analysis revealed that the total hardness of the mold-cooling water exceeded the standard, reaching 24 mg/L, which caused scaling on the mold copper plates and uneven cooling, leading to the frequent occurrence of longitudinal cracks. After strictly controlling the water quality, the issue of longitudinal cracks was brought under control. The online application of the tracing method for the causes of longitudinal cracks has effectively improved efficiency in resolving longitudinal crack problems.

## 1. Introduction

During the continuous casting process, timely control of key parameters associated with the formation of quality defects in cast slabs through online traceability can enhance the stability of casting and rolling production [1,2,3]. At the initial stage of production of a continuous slab casting machine, longitudinal cracks occurred frequently, particularly with an increased probability during the production of peritectic steel Q235B, severely impacting the stability of the final product quality.

Traditional methods for tracing the causes of longitudinal crack formation through defect sample analysis [4,5], physical simulation, and numerical simulation [6,7] require experienced metallurgical experts to spend a considerable amount of time and effort beforehand to determine the thermal physical properties of molten steel at different temperatures, such as thermal conductivity, specific heat capacity, and viscosity, and to determine high-temperature mechanical properties like yield strength, elastic modulus, and thermal expansion coefficient. Moreover, the method of tracing the causes of defects by offline comparative analysis of process parameters, raw and auxiliary material composition, and equipment precision data by process technology management personnel has significant latency and can no longer meet the needs for real-time intelligent control in the continuous casting and rolling production process [8,9].

With the rapid development of artificial intelligence technology [10,11,12,13], the online traceability method for the causes of longitudinal cracks based on AI technology has become the focus of research and application. There are many types of traceability methods: the empirical rule traceability method utilizes monitoring rules to diagnose abnormalities in production process parameters and trace the causes of defect formation; the statistical analysis traceability method traces the causes of longitudinal crack formation by analyzing the distribution of parameters; the intelligent analysis traceability method first fits the relationship between longitudinal cracks and their influencing factors, then deduces the weight of influential factors and traces the causes of longitudinal crack formation.

To achieve online traceability of the causes of longitudinal cracks, a data collection module and a data preprocessing module were first developed based on the characteristics of the continuous casting process data. The data were then matched and stored in information groups identified by billet numbers, forming a dataset of billet samples. On this basis, a model for tracing the causes of longitudinal crack formation applicable to different application scenarios was established. Compared to traditional offline analysis methods, the online traceability method for the causes of billet quality defects is more real-time and has a wider range of use, effectively improving the efficiency of solving longitudinal cracks.

## 2. Data Collection

### 2.1. Factors Affecting Surface Longitudinal Cracks in Continuous Casting Slab

**Figure 1:**
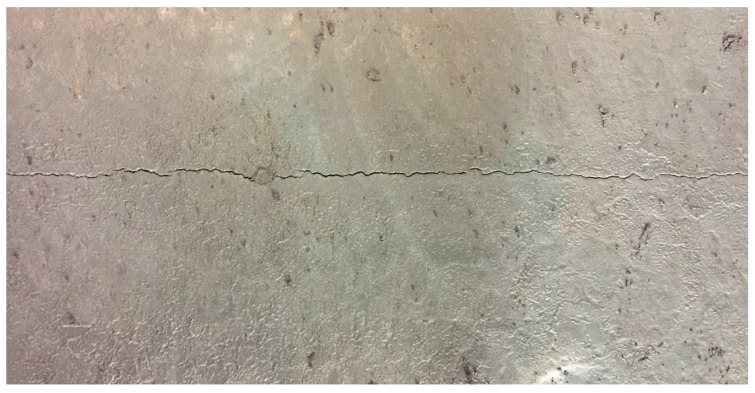
Surface longitudinal cracks in continuous casting slab.

Cracks in the billet can lead to minor cases requiring finishing treatment, while severe cases result in the scrapping of the billet [14,15], as shown in Figure 1. The main factors affecting longitudinal cracks include the following major categories [16,17,18,19]: (1) Factors characterizing the high-temperature mechanical properties of steel, including [C], [S], [Mn]/[S], and the crack sensitivity index of steel. (2) Factors characterizing the uniformity of mold cooling, such as the temperature difference between the inlet and outlet water of the mold, the heat flux density of the mold, the heat flux ratio between the narrow and wide faces of the mold, the difference in heat flux density across the symmetrical faces of the wide side of the mold, and the difference in heat flux density across the symmetrical faces of the narrow side of the mold. (3) Factors characterizing the adaptability of mold flux, such as the thickness of the liquid slag layer of the mold flux, the basicity of the mold flux, and the viscosity of the mold flux. (4) Factors characterizing the rationality of the mold flow field, including mold level fluctuation, stopper rod position, and depth of submersion in SEN. (5) Factors characterizing the precision of mold vibration, including vibration frequency, amplitude, and vibration deviation. (6) Factors characterizing equipment precision, such as the taper of the narrow face of the mold, the deviation between the center of the submerged nozzle and the center of the mold, and the diagonal deviation of the mold. (7) Abnormal operations, including nozzle change operations, open casting, submerged nozzle blockage, and slag line change. (8) Other key factors, including casting speed, casting speed fluctuations, liquid steel superheat, foot roller water spray intensity and zero segment water spray intensity.

During the continuous casting process, data on factors affecting longitudinal cracks are mainly obtained through online collection, online soft measurement, and manual offline input methods. The inflow temperature of the mold, the position of the stopper rod, and the insertion depth of the submerged entry nozzle are acquired through online collection. Parameters such as the heat flow ratio between the narrow left side and the wide inner side of the mold, the superheat of the molten steel, the negative slip time, and the steel’s crack sensitivity index are obtained through mechanism models or empirical formulas via online soft measurement. Parameters such as the operation of changing the submerged entry nozzle, blockage of the submerged entry nozzle, and the thickness of the protective liquid slag layer are acquired through manual offline detection by operators.

### 2.2. Data Integration Matching

#### 2.2.1. Data Collection and Preprocessing

**Figure 2:**
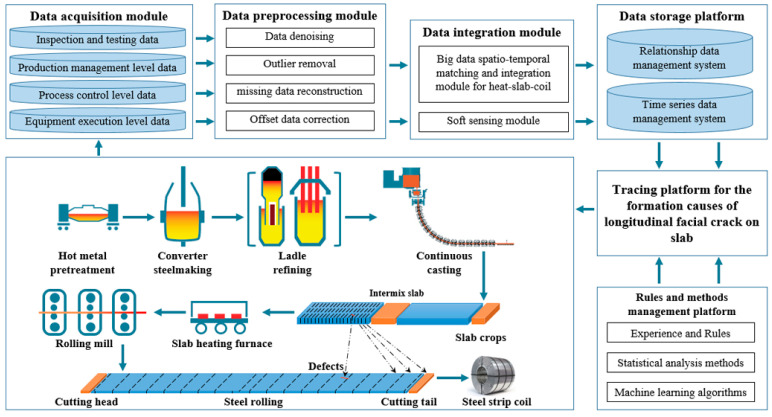
Online tracing process for the causes of longitudinal crack formation.

There are numerous factors affecting longitudinal cracks, and the complete and accurate collection and integration of these factors with feedback from crack inspections are fundamental for achieving online traceability. To ensure data integrity, a secure and stable data interface was constructed, connecting equipment at the execution, process control, production management levels, and the inspection and analysis systems across different stages of the steel manufacturing process to enable automatic data collection. After the completion of data collection, high-frequency data is stored in a time-series data management system, while low-frequency data is stored in a relational data management system, as shown in Figure 2.

**Figure 3:**
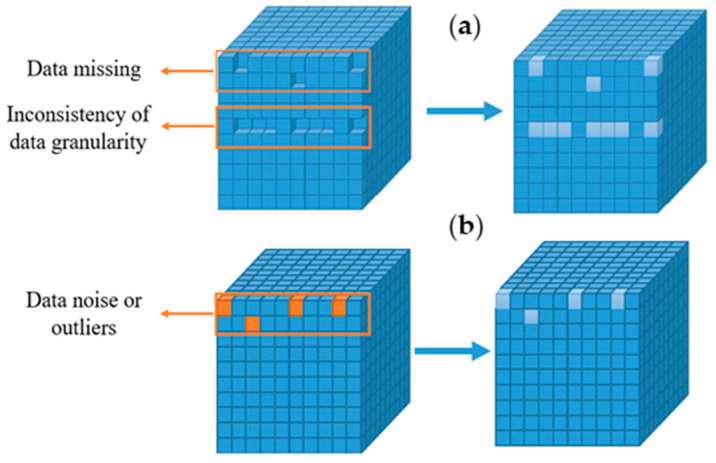
Data preprocessing. ((**a**) Handling of missing data; (**b**) handling of data anomalies).

In order to ensure data accuracy, preprocessing is conducted on missing data due to different collection frequencies, inconsistency in granularity, and issues in the continuous casting process data [20,21], as shown in Figure 3a [22]. Due to measurement errors and human mistakes, there is noise or outliers in the data, and data denoising and outlier removal must be performed [23,24], as shown in Figure 3b. The missing cubes in Figure 3a represent missing data caused by inconsistent collection frequency, granularity, or other reasons. The yellow cubes in Figure 3b represent noise data and outliers. These data are repaired through data preprocessing to ensure data integrity and reliability, as shown by the light blue cubes on the right side of Figure 3.

#### 2.2.2. Spatiotemporal Matching and Integration of Data

**Figure 4:**
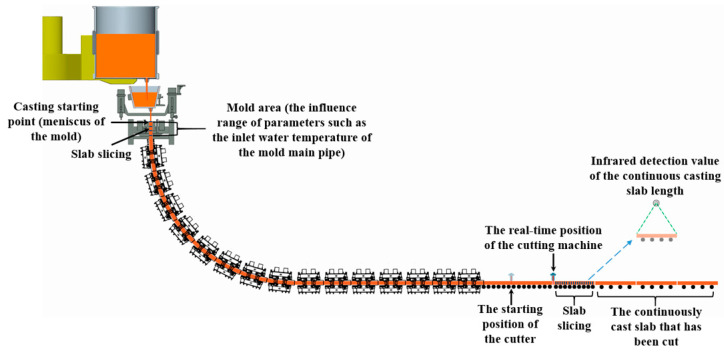
Data spatiotemporal matching.

After data preprocessing, the data is matched to the slab and slab slice information group, as shown in Figure 4.

**Figure 5:**
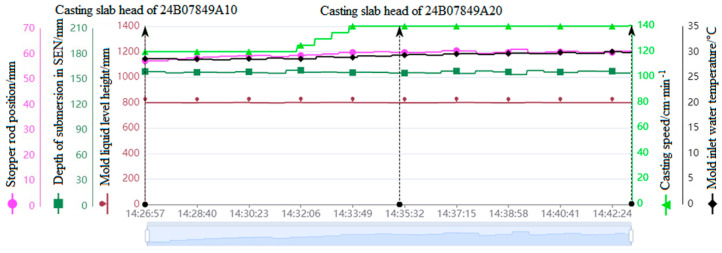
High−frequency parameters and casting space−time matching results.

The data matching process for the continuous casting process includes the following steps: (1) Determine the affected area for the parameters to be collected. For instance, the affected area for the mold inlet water temperature is the mold region. Based on the equipment drawings, the starting point of the mold region is set as the meniscus, and the endpoint is the mold exit. (2) Real-time recording of casting length: After the casting begins, the billet continuously solidifies from the crescent surface, and the casting length increases continuously. The casting length values at different times are recorded in real time. (3) Divide the entire casting length into fixed-length strand slices. As the casting progresses and the casting length increases, the strand continuously moves in the withdrawal direction, and the strand slices also move accordingly. (4) During the casting process, record the spatial positions of each strand slice in real time, along with the timestamps when each strand slice enters and exits the parameter-affected area. (5) Collect high-frequency parameters in real time, with a sampling frequency of 1 s. Based on the timestamps when the strand slices enter and exit the parameter-affected area, associate the high-frequency parameters within these time ranges with the corresponding strand slices. (6) After the strand is cut, determine the strand slices contained within the strand based on the spatial positions of the strand slices along the entire casting length. (7) Associate the high-frequency parameters with the strand based on the strand, strand slices, and the timestamps when the strand slices enter and exit the high-frequency parameter-affected intervals, as illustrated in Figure 5.

Figure 5 shows the correlation results online of the high-frequency parameter rod position, immersion nozzle insertion depth, mold liquid level height, pulling speed, and mold water manifold temperature with the slab, where the horizontal axis represents the time information and the vertical axis represents the scale information of high-frequency parameters. In addition, the upward arrows in Figure 5 indicate the moments when the slab enters and exits the mold area. For instance, the slab with the number 24B07849A10 entered the mold area at 14:26:57 and left at 14:35:25.

Based on this foundation, according to the spatiotemporal matching correlation results between the billet and high-frequency parameters, calculate the integrated characteristic values for each billet and each high-frequency parameter, as shown in Equations (1)–(4). Combined with the feedback results from the longitudinal crack inspection, analyze the characteristic values of the high-frequency parameters of the billet to achieve online tracing of the causes of continuous longitudinal crack formation.

(1) $$x_{mean}=\frac{1}{n}\sum_{i=1}^{n}x_{i}$$

(2) $$x_{max}=Max\left\{x_{1},x_{2},\ldots ,x_{n}\right\}$$

(3) $$x_{min}=Min\left\{x_{1},x_{2},\ldots ,x_{n}\right\}$$

(4) $$x_{\sigma }=\sqrt{\frac{1}{n}\sum_{i=1}^{n}{\left(x_{i}-x_{mean}\right)}^{2}}$$

In the formula, *x*_1_, *x*_2_, ⋯, *x_n_* are the *n* values collected for a certain parameter in the casting slice information group, *Max* is the maximum value solving function, *Min* is the minimum value solving function, *x_mean_* is the arithmetic mean, *x_max_* is the maximum value, *x_min_* is the minimum value, and *x_σ_* is the standard deviation.

## 3. Methods

### 3.1. Empirical Rule Traceability Method

The empirical rule traceability method uses pre-formulated monitoring rules to trace the weight order of the key influencing factors of longitudinal crack formation. In order to monitor the parameter stability in the production process of billet casting, this research team artificially formulated the billet parameter monitoring rules for steel number Q235B on the basis of statistical analysis of billet casting data of a steel enterprise and combined with the actual situation on site, as shown in Table 1.

The empirical rules consist of the lower control limit (*LCL*), lower warning limit (*LWL*), upper warning limit (*UWL*), and upper control limit (*UCL*). In the continuous casting production process, the influence weight of a certain factor is determined using Formulas (5)–(7).

(5) $$H_{1}=\frac{1}{n}\sum_{i=1}^{n}\sqrt{{\left(x_{i}-\frac{LWL+UWL}{2}\right)}^{2}}$$

(6) $$H_{0}=\frac{1}{m}\sum_{j=1}^{m}\sqrt{{\left(x_{j}-\frac{LWL+UWL}{2}\right)}^{2}}$$

(7) $$D=\frac{H_{1}}{H_{0}}$$

In the formula, *n* is the number of billet samples with longitudinal cracks; $x_{i}$ is the data value of the influencing factor for the *i*-th sample; m is the number of billet samples without longitudinal cracks; $x_{j}$ is the data value of the influencing factor for the *j*-th sample; $H_{E;1}$ is the stability index statistical value of a certain factor during the production process of billets with longitudinal cracks; $H_{E;0}$ is the stability index statistical value of a certain factor during the production process of billets without longitudinal cracks; $D_{E}$ is the result of the influence weight analysis.

### 3.2. Statistical Analysis Traceability Method

#### 3.2.1. Statistical Distance Analysis Method

**Figure 6:**
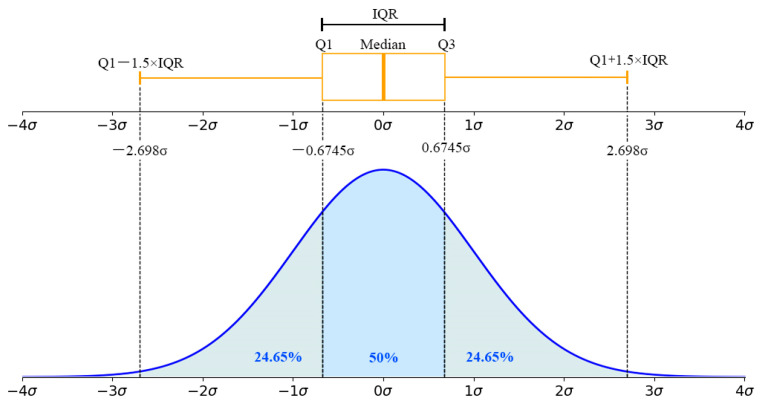
Normal distribution chart and box plot.

During the continuous casting production process, the changes in most parameters have statistical regularity, and low-probability events usually do not occur during a single monitoring. Once a low-probability event occurs in the change in a certain parameter, it indicates that the parameter is abnormal. In the continuous casting production process, the idea of statistical inference can be used to analyze the abnormal conditions of process parameters; when parameters fall outside the range of *μ* ± 3*σ*, they can be considered abnormal [25,26], as shown in Figure 6.

Based on the idea of statistical inference, the influence weight of each factor can be analyzed by calculating the statistical distance from sample data to the overall center, as shown in Formulas (8)–(10).

(8) $$H_{s,1}=\frac{1}{n}\sum_{i=1}^{n}\sqrt{{\left(\frac{x_{i}-\mu }{\sigma }\right)}^{2}}$$

(9) $$H_{s,0}=\frac{1}{m}\sum_{j=1}^{m}\sqrt{{\left(\frac{x_{j}-\mu }{\sigma }\right)}^{2}}$$

(10) $$D_{s}=\frac{H_{1}}{H_{0}}$$

In the formula, *n* is the number of longitudinal crack slab samples;$x_{i}$ is the influencing factor value of the *i*-th sample; m denotes the number of samples of cast billets without longitudinal cracks; $x_{j}$ is the corresponding value of the influencing factor for the *j*-th sample; $H_{s,1}$ is the stability index statistical value of a certain factor in the production process of slabs with longitudinal cracks; $H_{s,0}$ is the stability index statistical value of a certain factor in the production process of slabs without longitudinal cracks; and $D_{s}$ is the result of the influence weight analysis.

#### 3.2.2. Correlation Analysis Method

The correlation analysis method determines the weight of factors by analyzing the correlation coefficient between the longitudinal crack and each influencing factor [27,28], as shown in Formula (12):

(11) $$X=\left[\begin{array}{cccc} x_{11} & x_{12} & \ldots & x_{1p}\\ x_{21} & x_{22} & \ldots & x_{2p}\\ \vdots & \vdots & \ddots & \vdots \\ x_{n1} & x_{n2} & \ldots & x_{np} \end{array}\right],y=\left[\begin{array}{c} y_{1}\\ \begin{array}{c} y_{2}\\ \vdots \end{array}\\ y_{n} \end{array}\right]$$

(12) $$r_{j}=\frac{\sum_{k=1}^{n}\left(x_{kj}-\bar{x_{j}}\right)\left(y_{k}-\bar{y}\right)}{\sqrt{\sum_{k=1}^{n}{\left(x_{kj}-\bar{x_{j}}\right)}^{2}}\sqrt{\sum_{k=1}^{n}{\left(y_{k}-\bar{y}\right)}^{2}}}$$

In the formula, $y_{i}$ represents the feedback result of the longitudinal crack inspection, indicating whether a longitudinal crack exists or not. $\bar{x_{j}}=\frac{1}{n}\sum_{k=1}^{n}x_{kj}$,$\bar{y}=\frac{1}{n}\sum_{k=1}^{n}y_{k}$, $r_{j}$ represents the weight of the *j*-th factor.

Using the billet sample dataset, calculate the correlation coefficient between each influencing factor and the longitudinal crack feedback results. After sorting the correlation coefficients, obtain the order of importance of the influencing factors.

#### 3.2.3. Principal Component Analysis Method

Principal component analysis first combines the original variables into uncorrelated principal component variables through orthogonal transformation [29,30,31], and then determines the weights of the original variables by back-calculating using the variance contribution rate of the principal components. The steps are as follows:

(1) Standardize *X* to obtain matrix *Z*, and calculate the correlation coefficient matrix *R* = $\frac{Z^{T}Z}{n-1}$ of *Z* and the eigenvalues of *R*, resulting in p eigenvalues $\lambda_{1}\geq \lambda_{2}\geq \cdots \geq \lambda_{p}\geq 0$.
(2) Use the formula $\frac{\sum_{j=1}^{m}\lambda_{j}}{\sum_{j=1}^{p}\lambda_{j}}\geq 0.9$ to determine the value of *m*; for each $\lambda_{j}$, solve the equation $Rb=\lambda_{j}b$ to obtain the unit vector, $b_{j}^{0}=b_{j}/\left\|b_{j}\right\|$.
(3) Use formula $\alpha_{j}=\frac{\lambda_{j}}{\sum_{j=1}^{p}\lambda_{j}}$ to determine the variance contribution rate of the principal components and calculate the *m*-th principal component scores for $z_{i}$. In the formula, $u_{ij}=z_{i}^{T}b_{j}^{0}$ represents the principal component vector of the i-th variable.

(13) $$U=\left[\begin{array}{c} u_{1}^{T}\\ \begin{array}{c} u_{2}^{T}\\ \vdots \end{array}\\ u_{p}^{T} \end{array}\right]=\left[\begin{array}{cccc} u_{11} & u_{12} & \ldots & u_{1m}\\ u_{21} & u_{22} & \ldots & u_{1m}\\ \vdots & \vdots & \ddots & \vdots \\ u_{p1} & u_{p2} & \ldots & u_{pm} \end{array}\right]$$

According to the variance contribution rate of principal components, the weight of the original variable is determined by using the formula $\omega_{k}=\frac{\sum_{j=1}^{m}u_{kj}\alpha_{j}}{\sum_{j=1}^{m}\alpha_{j}}$, and the weight of the original variable is normalized to obtain the actual weight value.

The traceability method based on statistical analysis has high requirements for sample data, especially in terms of sample size, data distribution, data quality, and variable independence. If the data does not meet these requirements, it can cause false positives. The traceability method based on intelligent analysis has relatively low requirements for sample data, especially in terms of data distribution and variable independence, and this method has become the focus of research and application.

### 3.3. Intelligent Analysis Traceability Method

Based on intelligent analysis, the traceability method first requires the use of the strong nonlinear fitting capabilities of intelligent analysis methods [32] to fit the nonlinear relationship between longitudinal cracks and their influencing factors, and then determine the influence weights through back-calculation [33,34]. LightGBM (Light Gradient Boosting Machine) is a machine learning algorithm based on the gradient boosting framework, whose principle is to build a collection of weak models, with each step attempting to reduce the gradient of the loss function. This iterative process constructs tree models through weighted samples and continuously adjusts and improves its predictions to increase accuracy. The process of LightGBM fitting nonlinear relationships between inputs and outputs is as follows:

(1) Determine the objective function. Given a dataset ($x_{i}$,$y_{i}$)_(*i* = 1)*^n^*, the goal is to find a function *F*(*x*) to minimize the loss function *L*(*y*,*F*(*x*)).
(2) Model representation. The gradient boosting model is represented as $F\left(x\right)=\sum_{m=1}^{M}{\beta_{m}h}_{m}\left(x\right)$, where $h_{m}\left(x\right)$ is the m-th decision tree, and $\beta_{m}$ is the corresponding weight. Additionally, the model $F_{0}\left(x\right)=arg\;\underset{\beta }{\min}\sum_{i=1}^{n}L\left(y_{i},\beta \right)$ is initialized.
(3) For the *m* iteration, the model residuals are calculated $r_{i,m}$, as shown in formula $r_{i,m}=-{\left[\frac{\partial L\left(y_{i},F\left(x_{i}\right)\right)}{\partial F\left(x_{i}\right)}\right]}_{F\left(x\right)=F_{m-1}\left(x\right)}$.
(4) Construct a histogram to discretize the value of each feature into k buckets, and count the sum of the number of samples and gradients in each bucket.
(5) Find the best split point, for each bucket, calculate the gain of the split point, the gain formula is as shown in Formula (14), where $G_{L}$ and $G_{R}$ represent the gradient of the left subtree and the right subtree, $H_{L}$ and $H_{R}$ represent the second-order gradient of the left subtree and the right subtree, *λ* is the regularization parameter, and *γ* is the penalty term for the number of leaf nodes.
  (14) $$Gain=\frac{1}{2}\left(\frac{G_{L}^{2}}{H_{L}+\lambda }+\frac{G_{R}^{2}}{H_{R}+\lambda }-\frac{{\left(G_{L}+G_{R}\right)}^{2}}{H_{L}+H_{R}+\lambda }\right)-\gamma$$
(6) Split the node using the best split point found, and update the model, as in the formula $F_{m}\left(x\right)=F_{m-1}\left(x\right)+{\beta_{m}h}_{m}\left(x\right)$, where $\beta_{m}$ can be determined by a line search.

The relationship model between longitudinal cracks and their influencing factors was mapped by the LightGBM algorithm, and the influence of model input on output was quantified by SHAP (SHapley Additive exPlanations). The SHAP method proposed by Lundberg is a popular machine learning interpretation framework in recent years, and the calculation formula is shown in Formula (15) [35].

(15) $$\emptyset_{i}\left(f\right)=\sum_{S\subseteq N\backslash \left\{i\right\}}\frac{\left|S\right|!\cdot \left(\left|N\right|-\left|S\right|-1\right)!}{\left|N\right|!}\cdot \left[f\left(S\cup \left\{i\right\}\right)-f\left(S\right)\right]$$

where ∅*_i_*(*f*) is the Shapley value of factor *i*, *N* is the set of all factors, *S* is a subset of all factors, {*i*} is the *i*-th factor, and *f*(*S*) is the predicted output of the model at a given subset of factors *S*.

## 4. Results

### 4.1. Empirical Rule Traceability Results and Statistical Distance Traceability Results

**Figure 7:**
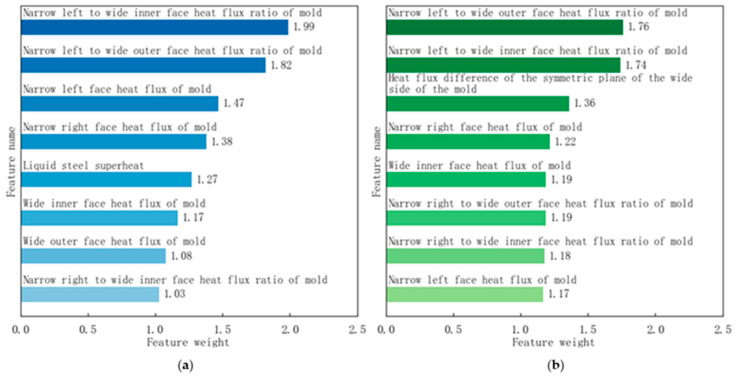
Traceability results based on distance: (**a**) empirical rule traceability results; (**b**) statistical distance traceability results.

During the initial production phase of a certain continuous caster, longitudinal cracks occurred frequently, especially during the production of Q235B killed steel, where the incidence rate reached 3.2%, severely affecting the stability of the casting and rolling process, as shown in Figure 2. The traceability results obtained through the empirical rule analysis method and the statistical distance analysis method are shown in Figure 7.

Empirical rule tracing results show that the heat flux ratio of the narrow left to wide inner face of the mold, heat flux ratio of the narrow left to wide outer face of the mold, and the heat flux of the narrow left face of the mold have significant impacts, with influence indices of 1.99, 1.82, and 1.47 respectively. Statistical distance tracing results indicate that the heat flux ratio of the narrow left to wide outer face of the mold, the heat flux ratio of the narrow left to wide inner face of the mold, and the heat flux difference of the symmetric plane of the wide side of the mold have significant impacts, with influence indices of 1.76, 1.74, and 1.36, respectively. Both methods point to uneven cooling of the mold as the main cause of frequent longitudinal cracking. The empirical rule tracing method and the statistical distance analysis tracing method are both distance-based analysis methods. For each factor, these methods first calculate the mean distance from numerous slab samples with longitudinal cracks to the target value, and then calculate the mean distance from numerous slab samples without longitudinal cracks to the target value. The ratio of these two means is used to quantify the weight order of the factors.

### 4.2. Correlation Analysis Results and Principal Component Analysis Results

**Figure 8:**
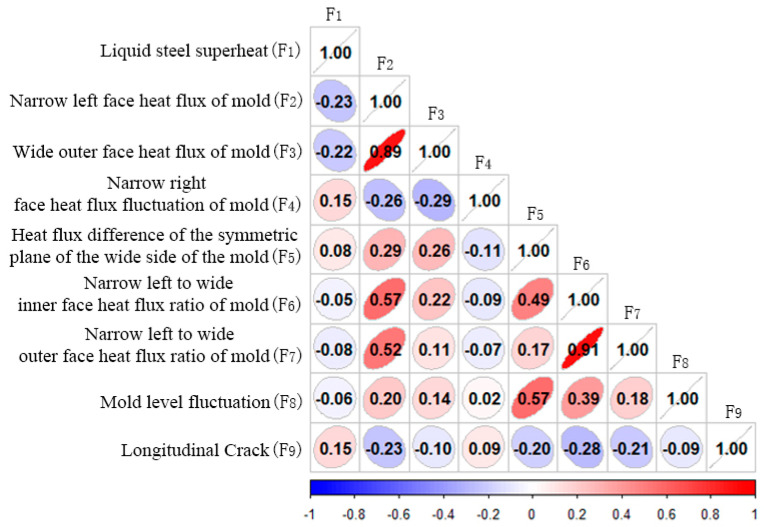
Correlation analysis result.

The correlation analysis results show that the heat flux ratio of the mold is narrow, the heat flux density of the mold is narrow, the heat flux ratio of the mold is relatively large, and the weights are −0.28, −0.23 and −0.21, respectively. The molten steel superheat is the second, with a weight of 0.15, as shown in Figure 8.

**Figure 9:**
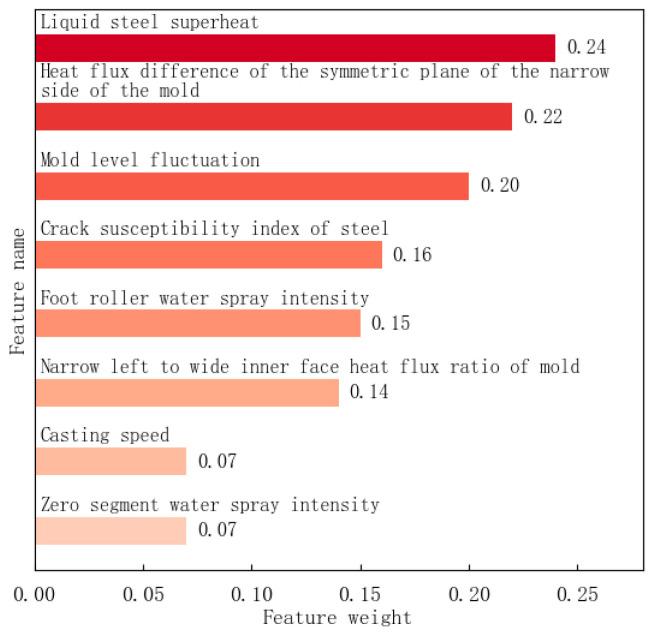
Principal component analysis result.

The results of the principal component analysis showed that the liquid steel superheat, the heat flux difference of the symmetric plane of the wide side of the mold, and the mold level fluctuation were relatively large, with weights of 0.24, 0.22 and 0.20, respectively, as shown in Figure 9. Different from the correlation analysis traceability method, the principal component analysis traceability method is an unsupervised traceability method, which does not need to be supported by longitudinal crack feedback results, and can be used in production scenarios with less longitudinal crack feedback data.

### 4.3. Intelligent Analysis Traceability Result

**Figure 10:**
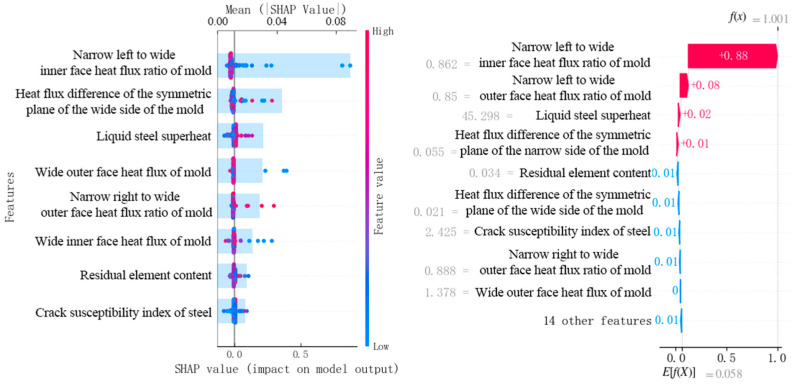
Traceability results of the LightGBM-SHAP method.

The LightGBM-SHAP traceability method was used to trace the influencing factors of the gating longitudinal crack, and the factors with greater influence are shown in Figure 10. In Figure 10, the left abscissa shows the ranking of the weights of the influencing factors, the specific weights refer to the histogram and the upper abscissa, and the results show that the heat flux ratio of the narrow left width of the mold is relatively greater, and the heat flux difference of the wide surface of the mold and the superheat of the molten steel are secondary. In addition, in Figure 10, each point represents the SHAP value of one sample in the slab sample dataset, the SHAP value magnitude is referenced to the lower abscissa, and each point shows the effect of the corresponding factors of each sample on the longitudinal crack. The red on the right ordinate represents a high value, the blue represents a low value, a red dot indicates that the value has a positive contribution to the longitudinal crack, and a blue dot indicates that the value has a negative contribution to the longitudinal crack. The analysis of Figure 10 shows that the heat flux ratio of the mold is too low to increase the probability of longitudinal cracks, and the high superheat of molten steel increases the probability of longitudinal cracks.

Figure 10 is a waterfall chart visualization of a single sample of a casting slab with longitudinal cracks based on SHAP explanations, showing how various factors gradually influence the formation of longitudinal cracks in the casting slab. *E*[*f*(*X*)] = 0.058 is the baseline value of the model, the average prediction output of the model, representing the overall prediction tendency of the model for all samples. Each row of the waterfall chart shows the contribution of each factor. Analysis of Figure 10 reveals that the longitudinal cracks in the casting slab are greatly influenced by the narrow left and wide inner heat flow of the mold.

## 5. Discussion

The results of the empirical rule traceability method, statistical distance traceability method, correlation analysis traceability method, principal component analysis traceability method and LightGBM-SHAP traceability method show that uneven cooling of the mold is an important reason for the high incidence of longitudinal cracks, as shown in Table 2.

**Figure 11:**
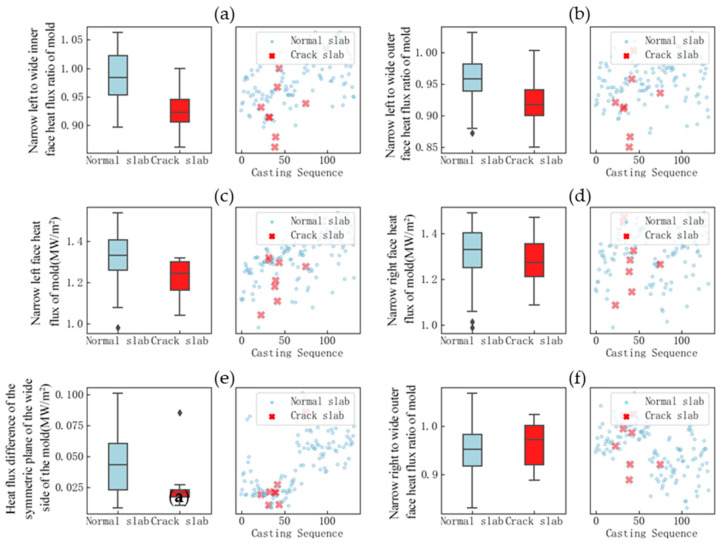
Distribution of factors characterizing uniform cooling of the mold: (**a**) narrow left to wide inner face heat flux ratio of the mold; (**b**) narrow left to wide outer face heat flux ratio of the mold; (**c**) narrow left face heat flux of the mold; (**d**) narrow right face heat flux of the mold; (**e**) heat flux difference of the symmetric plane of the wide side of the mold; (**f**) narrow right to wide outer face heat flux ratio of the mold.

For the cooling uniformity characterization parameters of the mold with great influence, the data differences between normal and longitudinal crack blanks were graphed and analyzed, as shown in Figure 11.

**Figure 12:**
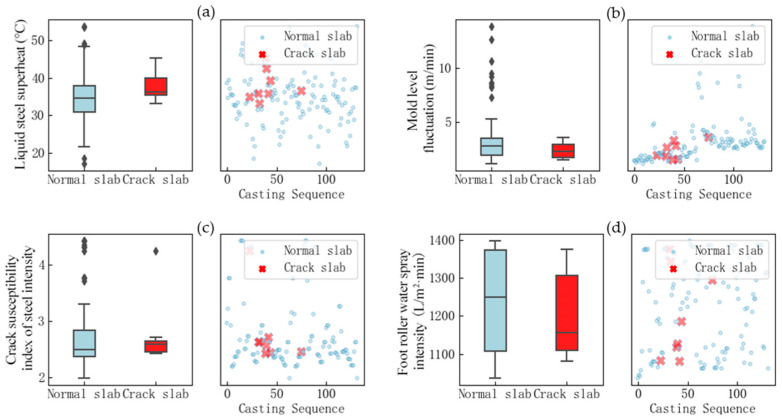
Comparative analysis of data distribution: (**a**) liquid steel superheat; (**b**) mold level fluctuation; (**c**) crack susceptibility index of steel intensity; (**d**) foot roller water spray intensity.

Analysis of Figure 11 shows that there are relatively large differences in the distribution of the narrow left width internal heat flow ratio, narrow left width external heat flow ratio, and narrow left heat flow density between normal billets and billets with longitudinal cracks. Uneven cooling of the mold increases the incidence of longitudinal cracks. In addition to the significant impact of uneven mold cooling, the results of the principal component analysis method indicate that superheat of molten steel, fluctuations in the mold liquid level, steel’s crack sensitivity, and the intensity of foot roller water spraying also have certain effects. Box plots and scatter plots were used for comparative analysis, as shown in Figure 12.

The analysis of Figure 12 shows that the superheat of the steel in the slab with longitudinal cracks is relatively high, while the data distribution of other factors shows little variation. This indicates that fluctuations in the mold liquid level, the crack sensitivity of the steel, and the intensity of foot roller water spraying are not the main influencing factors. Through analysis using different methods, it is known that the principal component analysis method is an unsupervised analysis method, which does not require feedback results from longitudinal cracks for support, leading to errors in the traceability results.

Combining the above results with the analysis of the mechanism of longitudinal crack formation, it is known that uneven cooling of the mold is the main reason for the longitudinal cracks in the slab of this continuous casting machine. When the cooling of the mold is uneven, coupled with the excessive overheating of the molten steel, it leads to frequent longitudinal cracks. During the continuous casting process of slabs, uneven cooling of the mold leads to uneven thickness of the slab shell. When the deformation at the weak points of the slab shell exceeds the critical strength and strain, longitudinal cracks form and propagate. When the liquid steel superheat is too high, the solidification of the molten steel is delayed, leading to a reduction in the thickness of the slab shell, which exacerbates the tendency for longitudinal cracking.

The slab longitudinal crack traceability method based on LightGBM-SHAP does not require the prior formulation of empirical rules compared to empirical rule traceability methods. This method utilizes the strong fitting capability of the LightGBM algorithm and the strong explanatory power of the SHAP algorithm to quickly fit the nonlinear relationship between longitudinal cracks and their influencing factors under unknown data distributions, demonstrating strong adaptability. The LightGBM-SHAP traceability method is not only suitable for the comprehensive traceability of multiple slab longitudinal cracks but also for the traceability of single slab longitudinal cracks, as shown in Figure 10. The LightGBM-SHAP traceability method provides a detailed explanation of the impact of each value of each factor on the slab longitudinal cracks, offering strong interpretability, as each scatter point shown in Figure 10 represents the specific impact on the longitudinal cracks. Therefore, this method is used for online traceability of the causes of longitudinal cracks. In the actual production process, the technicians carried out emergency handling by adjusting the cooling water flow rate of the mold wide face from 2850 L/min to 2750 L/min. By reducing the cooling water flow rate of the mold wide face, the heat flux density of the wide face was reduced, improving the uniformity of mold cooling, and thereby reducing the recurrence rate of longitudinal cracks. Root cause analysis found that the total hardness of the mold-cooling water exceeded the standard, reaching 24 mg/L, far exceeding the standard value of 10 mg/L. The substandard quality of the mold-cooling water caused scaling on the mold copper plate, leading to uneven cooling of the mold and frequent longitudinal cracks. After strictly controlling the water quality of the mold-cooling water, the longitudinal cracks were controlled, and the occurrence rate was reduced to 0.6%.

## 6. Conclusions

Research has developed empirical rule tracing methods, statistical distance tracing methods, correlation analysis tracing methods, principal component analysis tracing methods, and LightGBM-SHAP tracing methods. The principal component analysis tracing method is an unsupervised tracing method, which does not require feedback results from longitudinal cracks for support, leading to certain errors in tracing results. The other methods are supervised tracing methods, which can be applied according to the application scenario using the appropriate tracing method.

The LightGBM-SHAP tracing method, compared to empirical rule-based tracing methods, does not require the prior formulation of empirical rules, and demands lower requirements regarding data distribution and variable independence compared to statistical analysis tracing methods. This method effectively utilizes the strong fitting capabilities of the LightGBM algorithm and the strong explanatory power of the SHAP algorithm. It can rapidly fit the nonlinear relationship between longitudinal cracks and their influencing factors under unknown data distributions, demonstrating strong adaptability. This method is not only applicable to the comprehensive tracing of longitudinal cracks in multiple billets but also suitable for tracing in individual billets, providing detailed explanations of the impact of each value of each factor on the billet cracks, thus offering strong interpretability. Therefore, the LightGBM-SHAP method is used for online tracing of the causes of longitudinal crack formation.

During the initial production phase of a certain continuous slab casting machine, longitudinal cracks occurred frequently. The tracing results of the LightGBM-SHAP source tracing method indicated that the relative impact of the narrow left width internal heat flow ratio in the mold was relatively significant, followed by the heat flow difference on the wide face symmetrical surface of the mold and the superheat of the molten steel, with weights of 0.135, 0.066, and 0.048, respectively. Analysis revealed that uneven cooling of the mold was the cause of the frequent cracking. Process personnel urgently addressed this by reducing the cooling water flow on the wide face of the mold to improve cooling uniformity, thereby reducing the recurrence rate of longitudinal cracks. Further analysis found that the root cause of the frequent longitudinal cracks was the excessive total hardness of the mold-cooling water, which reached 24 mg/L, far exceeding the standard value of 10 mg/L. The application of the longitudinal crack formation source tracing method online plays an important role in enhancing the efficiency of solving longitudinal cracks.

## Figures and Tables

**Table 1:** Continuous casting production process parameter monitoring rules.

Parameter Name	LCL	LWL	UWL	UCL
Crack susceptibility index of steel	0	0	3	3.5
Residual element content (%)	0	0	0.038	0.042
Liquid steel superheat (°C)	12	15	30	33
Mold narrow face heat flux density (MW/m^2^)	1.25	1.3	1.4	1.45
Mold wide face heat flux density (MW/m^2^)	1.2	1.28	1.42	1.5
Mold heat flux fluctuation (MW/m^2^)	0	0	0.04	0.06
Symmetric face heat flux difference of mold (MW/m^2^)	0	0	0.06	0.08
Narrow to wide face heat flux ratio of mold	0.94	0.97	1.03	1.06
Casting speed (m/min)	0.85	0.9	1.05	1.1
Casting speed variation (m/min)	0	0	0.01	0.02
Mold level fluctuation (mm)	0	0	3	5
Depth of submersion in SEN (mm)	125	130	140	145
Mold flux liquid slag layer thickness (mm)	8	10	15	17
Mold flux consumption (kg/t)	0.4	0.45	0.6	0.7
Negative strip time ratio (%)	22.5	23	24	24.5
Foot roller water flow density (L/m^2^·min)	1100	1150	1300	1350
Zero segment water flow density (L/m^2^·min)	330	360	460	490

**Table 2:** Comparison of results from different traceability methods.

	Traceability Methods and Factor Weight Sequencing				
	Rule Traceability	Statistical Analysis Traceability	Correlation Analysis Traceability	Principal Component Analysis Traceability	LightGBM-SHAP Traceability
Narrow left to wide inner face heat flux ratio of mold	1/1.99	2/1.74	1/−0.28	6/0.14	1/0.089
Heat flux difference of the symmetric plane of wide side of mold	17/0.78	3/1.36	4/−0.20	2/0.22	2/0.044
Liquid steel superheat	5/1.27	16/0.72	5/0.15	1/0.24	3/0.031
Wide outer face heat flux of mold	7/1.08	9/1.07	6/−0.10	23/0.01	4/0.031
Narrow right to wide outer face heat flux ratio of mold	13/0.85	6/1.19	9/0.08	18/0.03	5/0.028
Wide inner face heat flux of mold	6/1.17	5/1.19	17/−0.04	10/0.06	6/0.024
Residual element content	10/1.01	13/0.85	20/−0.01	11/0.06	7/0.02
Crack susceptibility index of steel	9/1.02	15/0.78	13/0.06	4/0.16	8/0.019
Narrow left face heat flux of mold	3/1.47	8/1.17	2/−0.23	22/0.01	9/0.013
Narrow left to wide outer face heat flux ratio of mold	2/1.82	1/1.76	3/−0.21	9/0.06	10/0.012
Narrow right face heat flux of mold	4/1.38	4/1.22	12/−0.076	16/0.04	11/0.012
Narrow right face heat flux fluctuation of mold	16/0.81	22/0.52	7/0.09	17/0.03	14/0.009
Casting speed	11/0.99	12/0.99	11/−0.07	7/0.07	16/0.006
Narrow right to wide inner face heat flux ratio of mold	8/1.03	7/1.18	21/0.01	14/0.05	17/0.006
Mold level fluctuation	22/0.50	18/0.69	8/−0.09	3/0.2	18/0.004
Foot roller water spray intensity	12/0.98	10/1.01	14/−0.067	5/0.15	20/0.001
Zero segment water spray intensity	19/0.72	14/0.84	15/−0.05	8/0.07	21/0.001

## Data Availability

The original contributions presented in this study are included in the article, further inquiries can be directed to the corresponding author.
